# Loss of Parafollicular Cells during Gravitational Changes (Microgravity, Hypergravity) and the Secret Effect of Pleiotrophin

**DOI:** 10.1371/journal.pone.0048518

**Published:** 2012-12-19

**Authors:** Elisabetta Albi, Francesco Curcio, Renza Spelat, Andrea Lazzarini, Remo Lazzarini, Samuela Cataldi, Elisabetta Loreti, Ivana Ferri, Francesco Saverio Ambesi-Impiombato

**Affiliations:** 1 Laboratory of Nuclear Lipid BioPathology, Centro Ricerche Analisi Biochimico Specialistiche, Perugia, Italy; 2 Department of Clinical and Biological Sciences, University of Udine, Udine, Italy; 3 Institute of Pathologic Anatomy and Histology, University of Perugia, Perugia, Italy; Baylor College of Medicine, United States of America

## Abstract

It is generally known that bone loss is one of the most important complications for astronauts who are exposed to long-term microgravity in space. Changes in blood flow, systemic hormones, and locally produced factors were indicated as important elements contributing to the response of osteoblastic cells to loading, but research in this field still has many questions. Here, the possible biological involvement of thyroid C cells is being investigated. The paper is a comparison between a case of a wild type single mouse and a over-expressing pleiotrophin single mouse exposed to hypogravity conditions during the first animal experiment of long stay in International Space Station (91 days) and three similar mice exposed to hypergravity (2Gs) conditions. We provide evidence that both microgravity and hypergravity induce similar loss of C cells with reduction of calcitonin production. Pleiotrophin over-expression result in some protection against negative effects of gravity change. Potential implication of the gravity mechanic forces in the regulation of bone homeostasis via thyroid equilibrium is discussed.

## Introduction

Parafollicular cells or Thyroid C cells are generally known for producing calcitonin, a hormone involved in calcium homeostasis with hypocalcemic and hypophosphatemic effects but it has been highlighted their role in the production of numerous regulatory peptides such as somatostatin and ghrelin [1], katacalcin I, katacalcin II, gastrin-releasing peptide, thyroliberin and helodermin [2]. Moreover C cells, under regulation by thyrotropin (TSH) because of TSH receptor (TSHR) expression, are involved in the hypothalamic-pituitary-thyroid axis [3]. Accumulating evidence showed that C-cells express thyrotropin releasing hormone (TRH) carrying out paracrine activity on follicular cells and inducing in them TRH-Rs expression [4]. In this way C cells are responsible for intrathyroidal regulation of follicular cells by permitting an interrelationship between the two endocrine populations [5].

Studies on the behavior of the thyroid C cells in follicular pathological conditions are contradictory. Maternal hypothyroidism induced by ^131^I leaded to the development of hyperplasia and hyperthrophy of calcitonin-positive cells in the pups at the time of birth [6]. Differently, hypothyroidism evoked by propylthiouracil attenuated density of parafollicular cells [7]. In addition less numerous C cells were found in simple and hyperactive goitre in comparison with normal thyroid parenchyma while proliferative changes concerned only follicular cells [8]. It is possible that the variance of results was due to greater complexity of the intrathyroidal regulatory pathway involving several C cell functions.

Space missions are an excellent model to study the simultaneous changes in bone and follicular thyroid metabolism, both affected from C cells. In fact, spaceflight generated a skeletal adaptive response resulting in the loss of bone mass with the change of osteoblast differentiation and morphology [9], calcium metabolism and biochemical markers of bone turnover [10], bone formation and resorption processes [11]. Changes in blood flow, systemic hormones, and locally produced factors were indicated as important elements contributing to the response of osteoblastic cells to loading [9] but research in this field still has many questions. It has been demonstrated that in the longest mice permanence (91 days) on International Space Station (ISS) during the Mice Drawer System (MDS) mission, animals presented a bone loss but transgenic mice over-expressing pleiotrophin (PTN-TG), molecule that produces positive effects on bone turnover, had an osteoblast activity higher than that observed in wild type (WT) mice, indicating that the expression of the PTN during the flight resulted in some protection against microgravity's negative effects [12]. In the same experimental model, the structure of thyroid follicles appeared more organized, TSHR more expressed, cAMP release under TSH stimulation more intense in spaceflight mice than in control animals. The thyroid of PTN-TG mice was characterized by poorly developed follicles that were heterogeneous because of the variable size of both cells and colloidal spaces and the variability increased strongly in space environment together to an increase of TSHR and cAMP although with lower values than those of WT mice [13]. In spite of the existence of data on thyroid follicular cells changes during space missions, no observation has ever been recorded on thyroid parafollicular cells in the space environment. Here we reported the results of the behavior of C cells obtained by using the same mice of the same experimental model of Tavella et al. [12] and Masini et al. [13] to understand their interaction with bone metabolism. To test the role of the physical force of gravity on the modifications obtained during the mission, the experiments were repeated in conditions of hypergravity.

## Results

### 1. How thyroid parafollicular cells sense the change of the gravity

**Figure 1:**
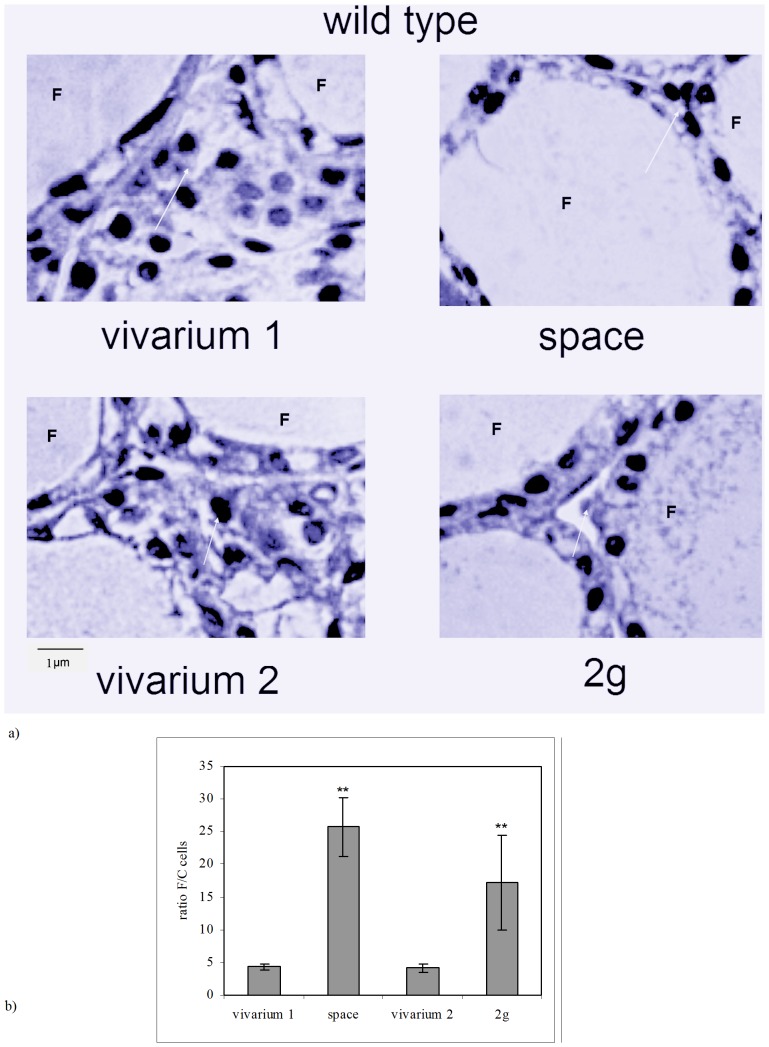
Effect of the gravity change on thyroid tissue of WT animals. a) Morphology analysis of parafollicular thyroid cells. “vivarium 1”: mice maintained in vivarium cages (control for experiment in hypogravity); “hypogravity”: experimental mouse in space; “vivarium 2”: control for experiment in hypergravity; “hypergravity”: experimental mice in 2×g centrifuge. Hematoxylin-eosin staining, 40× magnification, 1 µm scale bar. F = follicle. b) Ratio between the number of follicular cells of three follicles delimiting a parafollicular area and the number of cells C in this area. The values are expressed as mean ± SD of three independent fields observed in duplicate (7 and 13 sections). (Significance, **P<0.001 space versus vivarium 1 and 2 g versus vivarium 2).

**Figure 2:**
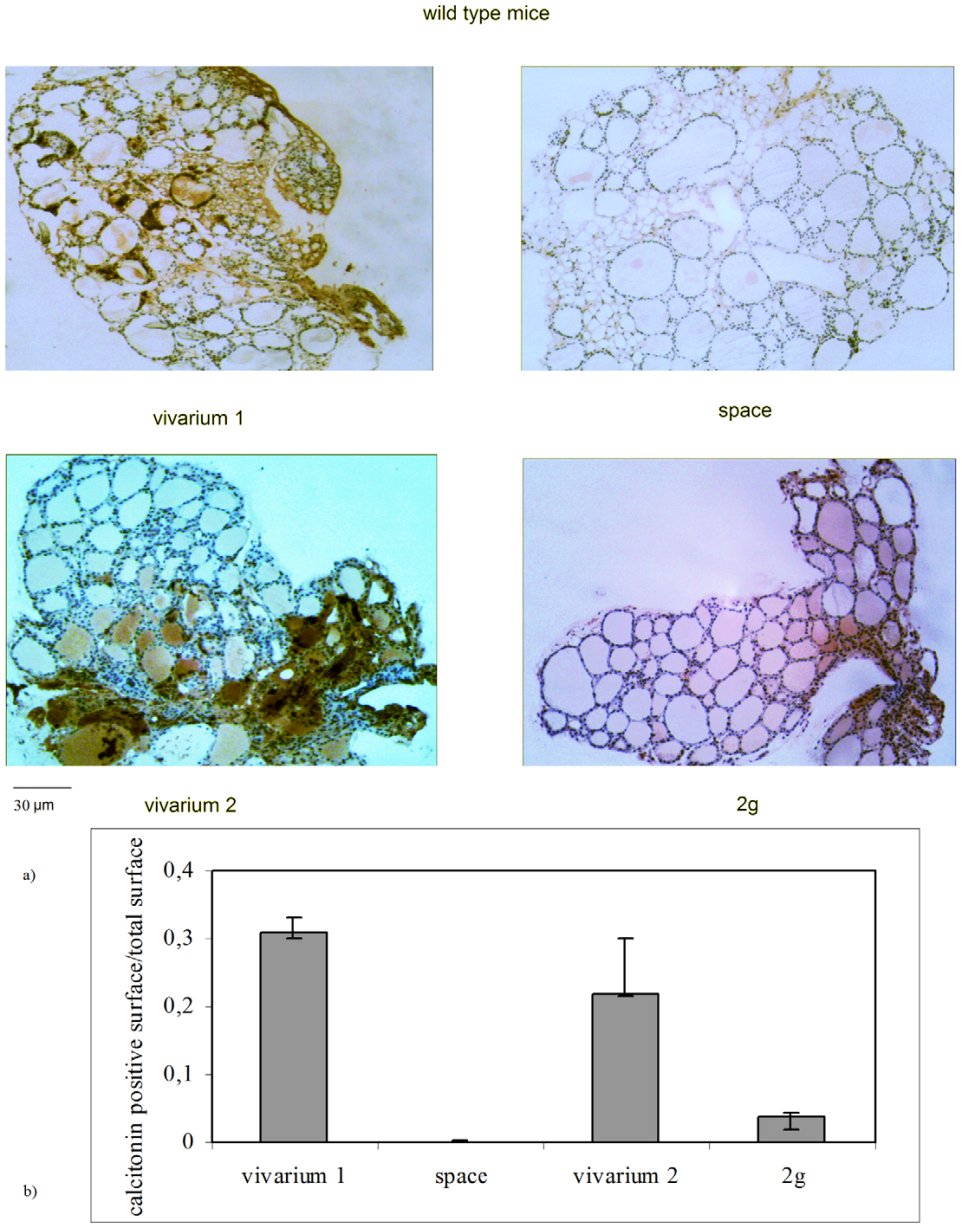
Effect of the gravity change on calcitonin production in WT animals. a) Calcitonin detection in thyroid tissue of WT animals. “vivarium 1”: mice maintained in vivarium cages (control for experiment in hypogravity); “hypogravity”: experimental mouse in space; “vivarium 2”: control for experiment in hypergravity; “hypergravity”: experimental mice in 2×g centrifuge. Immunohistochemical staining. 4× magnification, 30 µm scale bar. b) Ratio between the parafollicular surface and total surface of thyroid lobe. The values are expressed as median and range of two sections (8 and 14).

We have previously demonstrated that while in the thyroid gland of WT control mice the follicles had variable size and spatial orientation, spaceflight animals presented a more homogenous thyroid tissue structure, with ordered follicles and reduction of interfollicular space [14]. Since most species C cells are mainly concentrated in the middle third of each thyroid lobe, the so-called *C-cell region* [15], we have focused the attention on this specific area. Our observations showed that in this area each interfollicular space is delimited by three follicles. Fig. 1a shows the particular of the walls of two adjacent follicles normally structured with numerous interfollicular cells in vivarium 1 (control for the space experiment). It is known that the follicle is surrounded by thyrocytes or follicular cells. The analysis of the cell number in vivarium 1 sample highlighted that the sum of the follicular cells of three follicles delimitating an interfollicular space is 78±9 whereas the number of C cells is 18±3. The ratio between the two cell types is reported in Fig. 1b. In space environment the interfollicular space is strongly reduced (Fig. 1a) and the number of follicular and C cells is 75±6 and 3±2 respectively, by increasing consequently their ratio (Fig. 1b). Thus it is clearly evident that the space environment induces a loss of C cells. To try to discriminate whether this effect was due to the reduction of gravity force or to other factors of the space environment we thought to repeat the experiments in hypergravity condition with the idea of obtaining or opposite results for the principle of opposites or similar results. This would open a whole issue related to the fact that any change of a physical force of gravity would have an impact on cellular function. The results have highlighted that the number of follicular and C cells in the control sample (vivarium 2) is 66±8 and 16±7 respectively, similar to those of vivarium 1 and consequently they have a similar ratio (Fig. 1b). In hypergravity conditions the number of follicular and C cells is 69±9 and 4±3 respectively, by increasing their ratio with respect to vivarium 2 (Fig. 1b). If you compare the results of hypo- and hypergravity it appears evident that they induce a similar effect on the reduction of C cells. Since thyroid C cells are mainly known for producing calcitonin we have performed immunohistochemical analysis with anti-calcitonin antibodies to test C cell function. The results show the immunopositivity in the central regions of the thyroid gland lobes, as expected, of vivarium 1 and vivarium 2 controls (Fig. 2a). Median and range values of surface area are 3,49 (3.86–3,39) mm^2^ and 2,77 (3,45–2,71) mm^2^ in the vivarium 1 and vivarium 2 respectively. Either in space sample or in 2 g sample the immunopositivity is strongly reduced (Fig. 2a) even if with different values. In fact, in the space environment the immunopositivity is evident in a surface equal to 0,019 (0,015–0,021) mm^2^ whereas in 2 g sample the value of surface is 0,39 (0,37–0,43). The ratio between the value of immunopositivity surface and total surface of the thyroid lobe is reported in Fig. 2b. Even if the number of cells C is similar in hypo- and hypergravity, the surface of the positive area to anti-calcitonin antibody is wider in hyper- than in hypogravity. This result allows to suppose that the few cells present are more active in hyper- than in hypogravity.

### 2. Pleiotrophin keeps thyroid C cells in shape

Since the over expression of the PTN during the spaceflight resulted in some protection against microgravity's negative effects on bone tissue [12], we wanted to investigate whether there was an effect of this protein on thyroid parafollicular cells and the production of calcitonin involved in bone metabolism.

**Figure 3:**
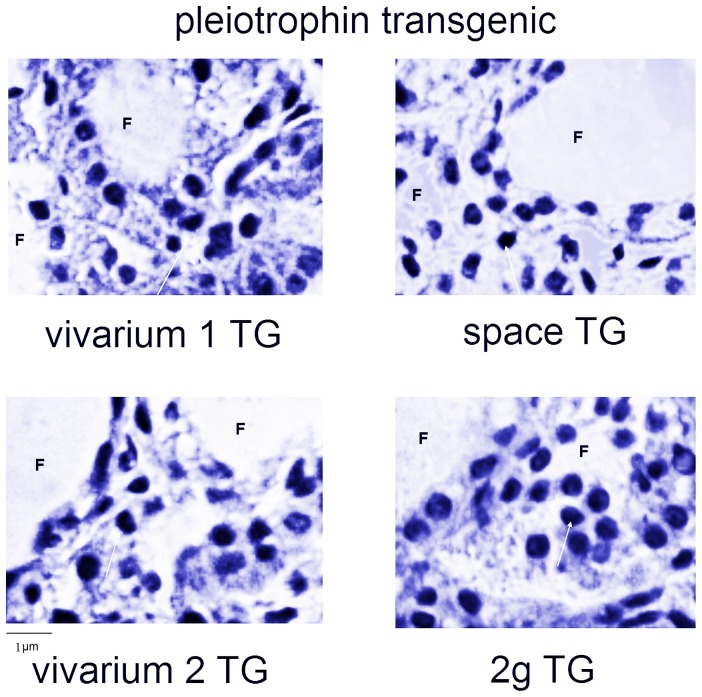
Effect of the gravity change on thyroid tissue of PTN-TG animals. Morphology analysis of parafollicular thyroid cells. “vivarium 1”: mice maintained in vivarium cages (control for experiment in hypogravity); “hypogravity”: experimental mouse in space; “vivarium 2”: control for experiment in hypergravity; “hypergravity”: experimental mice in 2×g centrifuge. Hematoxylin-eosin staining, 40× magnification, 1 µm scale bar, F = follicle.

**Figure 4:**
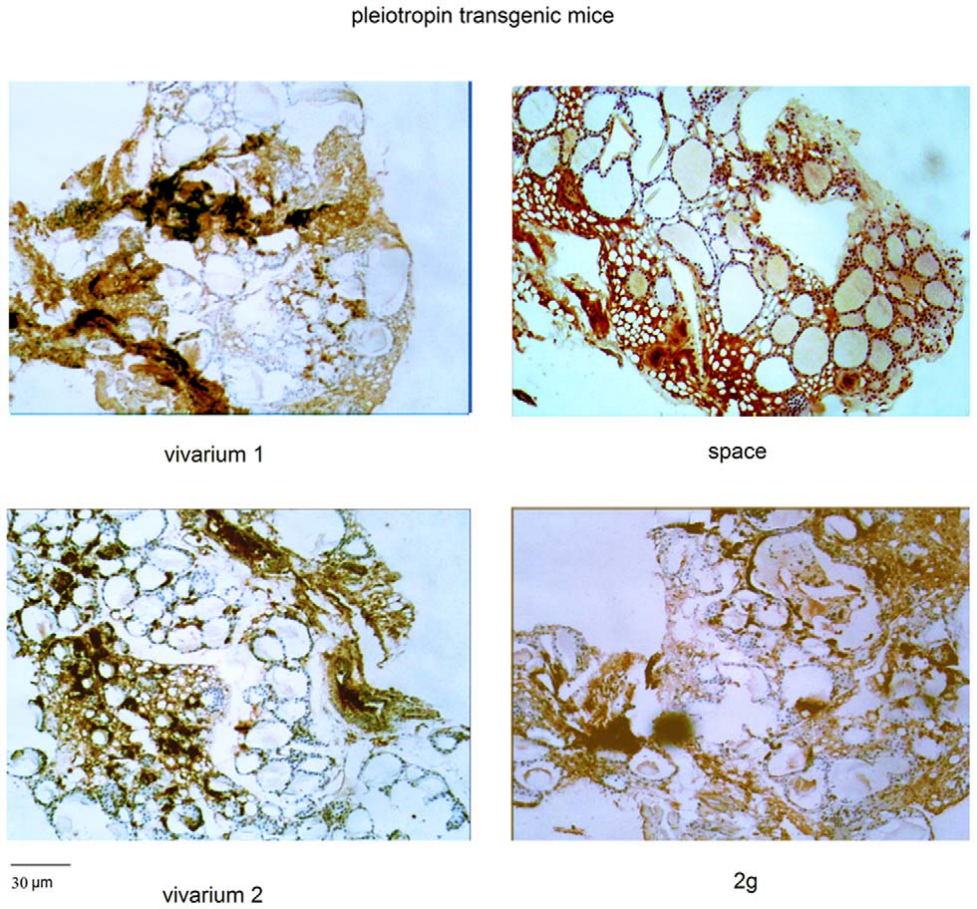
Effect of the gravity change on calcitonin production in WT animals. Calcitonin detection in thyroid tissue. “vivarium 1”: mice maintained in vivarium cages (control for experiment in hypogravity); “hypogravity”: experimental mouse in space; “vivarium 2”: control for experiment in hypergravity; “hypergravity”: experimental mice in 2×g centrifuge. Immunohistochemical staining. 4× magnification, 30 µm scale bar.

We previously noted that the thyroid of PTN-TG mice were characterized by follicles poorly developed and with variable size of thyrocytes and colloidal spaces [13], suggesting that the over-expression of PTN induces a follicular change. Here we show that the number of C cells is high in both control samples, vivarium 1 and vivarium 2, as occurs in WT mice thyroid, indicating that the PTN over-expression does not influence C cells in the ground (Fig. 3). Differently in both space and 2 g animals, the PTN over-expression reduces strongly the loss of C cells observed in WT mice thyroid. Because of the high irregularity of the thyroid lobe structure is really difficult to make an accurate analysis of the number of follicular and C cells. In fact, in Fig. 4, the vivarium 1 and vivarium 2 samples show follicles altered in shape and size with abnormal light areas with respect to WT samples (Fig. 2), supporting previous results [13]. The labelling for calcitonin is similar to that of WT samples. In the spaceflight animals the size of follicles is greatly heterogeneous with nuclei more evident, supporting previously observation [13] with the positivity for calcitonin lower than that of its control (vivarium 1) but significantly higher than that of WT mice. In this sample the immunopositivity is irregular and spread unevenly and it is very difficult to calculate the surface area occupied. In the 2 g sample you can see the similar results (Fig. 4).

## Discussion

In this study, we provide evidence that both microgravity and hypergravity induce similar numeric and functional changes of thyroid parafollicular cells, suggesting a potential implication of the mechanic forces in the regulation of bone homeostasis via thyroid equilibrium. It has been demonstrated that *in vitro* microgravity and hypergravity produce contrary effects. In fact, in Helix lucorum and Pomatias rivulare, statoconia and statoliths grew in number significantly in hypogravity whereas hypergravity caused their massive destruction [16]. Equally, global transcriptional state of Arabidopsis thaliana was influenced in opposite way in the two experimental models [17]. Platelet aggregation and platelet adhesion to von Willebrand factor were significantly decreased after platelets were exposed to simulated microgravity. Conversely, these platelet functions were increased after platelets were exposed to hypergravity [18]. It is possible to think that the effect of gravity changes on a complete (multicellular) organism, where multiple mechanisms of functional regulation are present, is more complex. In addition, in each tissue the balance between mechanical forces, both intra- and extracellular, that determine cell shape and integrity, influences and is influenced by what happens in neighboring cells. Very complex are the mechanisms of metabolic regulations. Bone loss is one of the most important complications for astronauts who are exposed to long-term microgravity in space. It is generally known that microgravity is associated with the loss of bone in astronauts resulting from an exceptional form of disuse together to mineral metabolism alteration and the loss of information that must be communicated to the effector cells that form new bone or destroy old bone mediated by different molecules such as prostaglandin and prostaglandin G/H synthase inducible cyclooxygenase [19]. And so, dependent on the mission length, individual turnover rates, stress, nutrition, fluid shifts, dehydration and bone perfusion, the astronauts undergo osteoporosis characterized by deterioration of bone tissue leading to enhanced bone fragility and to a consequent increase in fracture risk [20]. The development of space osteoporosis is supported by low energy intake, low calcium intake, low plasma 25-hydroxy-vitamin D or low calcitriol levels but dietary calcium and vitamin D do not stabilize bone turnover because markers of bone formation were reduced and markers of bone resorption were increased [21]. So far no data exist, obtained from space missions, regarding the production of calcitonin, a pharmacological inhibitor of osteoclastic bone resorption. And yet, nasal calcitonin treatment provided dual action on osteoporosis and osteoarthritis with significant improvements in quality of life [22].

Here we show the first evidence of the parafollicular cells loss and the reduction calcitonin production in the absence of weight. We propose that the reduction of calcitonin may be involved in osteoporotic damage produced during space missions. Our results clearly indicate that the hypergravity induces a similar effect to that obtained in hypogravity, suggesting that, in any case, the change of mechanical force results in a stress condition with the same damage on thyroid parafollicular cells. It has been demonstrated that short term exposure to hypergravity induces significant reductions in the thicknesses of cortical bone at the anterior (13%) and medial regions (15%) of the mid-diaphysis but femoral bone density, collagen and calcium concentrations are unaltered [23]. In addition the content of mature, stable bone collagen cross-links hydroxylysylpyridinoline (HP), lysylpyridinoline (LP), are significantly greater in bones from centrifuged animals than in bones from control animals [23]. Since it was not possible to measure the bone turnover markers in our study because of the unavailability of the blood of animals, we do not know at the time the effects on bone metabolism of long stay in hypergravity conditions.

Since the spatial integration of follicular and parafollicular cells and functional coordination of both epithelial cell lines exists in normal conditions [24], it is possible that modifications of follicular cells during space mission [13] and in hyper-gravity conditions, regulated in turn by hypothalamus, are responsible for parafollicular cell changes. The loss of calcitonin in hypergravity rather than act on bone metabolism may play a role in the intrathyroidal regulatory pathway of thyroid hormone synthesis. Here we report that over expression of PTN, or osteoblast-stimulating factor 1 or heparin-binding growth-associated molecule [25], limits the damage produced by hypo- or hypergravity conditions. Tavella et al. [12], discussed that during flight WT mice tend to lose more bone trabeculae than PTN-TG mice, suggesting that the over expression of the PTN exerts some protection on the skeleton against the bone loss consequent to the microgravity exposure but how PTN transgene could prevent in the transgenic mice bone tissue cell morphology alteration observed in WT bones is not defined. The authors shown that the reduction in the expression of collagen type I and osteocalcin in PTN-TG was less than in the samples from WT mice. We propose a reduction bone resorption due to the higher level calcitonin expression in PTN-TG mice in comparison with WT mice that could participate to the protective effect of PTN overexpression on the bone damage. To confirm our results it would be really important to know the blood levels of calcitonin in the hypogravity and hypergravity of WT or PTN-Tg mice but in this study we have participated in a “Tissue Sharing Program” in which every group has collected and studied the organ of his interest. We have taken the thyroids which were the subject of our study, while blood was collected from other groups for different analyses. Future space missions and hypergravity experiments could clarify this aspect of the study.

## Materials and Methods

### Experimental design and animal care

All experimental procedures were authorized by the Public Veterinary Health Department of the Italian Ministry of Health. The experiment was also conducted in accordance with the regulations for the care and use of laboratory animals and with the guidelines of the Japanese Physiological Society. Furthermore, this study was also approved by the Committee on Animal Care and Use at Graduate School of Medicine, Osaka University (No. 22-071). Finally, the protocol utilized in the study has been authorized by the Public Veterinary Health Department of the Italian Ministry of Health. All experiments were carried out using male C57BL/10J mice (8 weeks old).

### Hypogravity experiment

WT and PTN-TG mice (n = 3 each) were individually housed in the Mouse Drawer System (MDS), a 11.6×9.8×8.4 cm payload developed by Thales-Alenia Space Italy and all treatments were performed as previously reported [13]. Food and water were supplied *ad libitum*. The MDS, loaded with 3 WT and 3 TG mice, was launched in the Space Shuttle Discovery, within the Space Transport System (STS)-128 mission, on August 28, 2009. It was then housed in Japanese Experimental Module (Kibou) on the ISS until its return to the Earth by Space Shuttle Atlantis (STS-129 mission) on November 27, 2009. Only 1 WT and 2 TG mice returned to the Earth alive after 91 days of space flight.

Thyroids were sampled bilaterally from each mouse killed by inhalation of carbon dioxide at the Life Sciences Support Facility of Kennedy Space Center within 3–4 hours after landing and either processed or frozen immediately, according to the various experimental protocols. The procedure was approved by the IACUC protocol n° FLT-09-070(KSC).

After the spaceflight experiment, the on-ground experiment was also carried out at the Vivarium of the Advanced Biotechnology Center in Genova, Italy. One group of mice with the same species, sex, and age were housed in normal vivarium cage as the laboratory control (Vivarium1). Amount of food and water supplementation and environmental conditions were simulated as the flight group. After 3 months, thyroids were sampled bilaterally from 3 WT and 3 TG mice and treated as above reported for spaceflight mice.

### Hypergravity experiment

WT and PTN-TG mice (n = 3 each) of the same strain as those used in hypogravity experiments, were maintained in hypergravity, with conditions similar to the MDS experiment, in a 2×g centrifuge in the laboratory of Dr. Y. Ohira at the Osaka University, Osaka, Japan. Control mice were similar to those reported in hypogravity experiment (Vivarium 2). Animals were treated, and thyroids were obtained and processed with the same procedures used in the hypogravity/space experiments.

### Thyroid tissue treatment

The thyroid lobes were fixed in 4% neutral phosphate-buffered formaldehyde solution for 24 h as previously reported [13]. Thyroids were dropped with essentially random orientation in paraffin. The paraffin blocks were sectioned into 4-µm-thick sections. All sections were mounted on silan-coated glass slides. Each slide contained a pair of sections at a distance equal to 140 µm. Between 7 and 14 pairs of sections were sampled excluding the first and the last; 7 and 13 sections were used for morphological analysis whereas 8 and 14 sections were used for immunohistochemical analysis. Tissue sections were deparaffinized and rehydrated through a series of xylene and ethanol washes.

### Morphological analysis

The sections were stained by the hematoxylin-eosin (Chroma-Gesellschaft, Germany) staining method and investigated for parafollicular cells detection by using inverted microscopy EUROMEX FE 2935 (ED Amhem, The Netherland) equipped with a CMEX 5000 camera system (40× magnification).

### Immunohistochemical analysis

For immunohistochemical analysis Bond Dewax solution was used for removal of paraffin from tissue sections before rehydration and immunostaining on the Bond automated system (Leica Biosystems Newcastle Ltd, UK) as previously reported [14]. Immunostaining for calcitonin detection was performed according to Bancroft and Stevens [15] by using NCL-L-calcitonin and Bond Polymer Refine Detection - Leica Biosystems ((Newcastle Ltd, UK). The observations were performed by using inverted microscopy EUROMEX FE 2935 (ED Amhem, The Netherland) equipped with a CMEX 5000 camera system (4× magnification). The analysis of the tissue section size was performed by ImageFocus software.

### Statistical analysis

The experiments have been conducted on the thyroid of: 1 animal for the hypogravity experiment (the only returned alive from the mission), 3 control animals for the hypogravity experiment (vivarium 1); 3 animals for the hypergravity experiment; 3 control animals for the hypergravity experiment (vivarium 2). For morphological analysis, the means ± SD of 3 fields of the 7 and 13 sections were given. The significance of the differences between the data was checked by Student's t-test. For immunohistochemical analysis the medians and ranges of 8 and 14 sections were given.
